# Longitudinal OCT and OCTA monitoring reveals accelerated regression of hyaloid vessels in retinal degeneration 10 (rd10) mice

**DOI:** 10.1038/s41598-019-53082-9

**Published:** 2019-11-13

**Authors:** Tae-Hoon Kim, Taeyoon Son, David Le, Xincheng Yao

**Affiliations:** 1 Department of Bioengineering, University of Illinois at Chicago, Chicago, IL 60607 USA; 2 Department of Ophthalmology and Visual Sciences, University of Illinois at Chicago, Chicago, IL 60612 USA

**Keywords:** Neuronal development, Neurophysiology, Imaging and sensing

## Abstract

The hyaloid vascular system (HVS) is known to have an important role in eye development. However, physiological mechanisms of HVS regression and their correlation with developmental eye disorders remain unclear due to technical limitations of conventional ending point examination with fixed tissues. Here, we report comparative optical coherence tomography (OCT) and OCT angiography (OCTA) monitoring of HVS regression in wild-type and retinal degeneration 10 (rd10) mice. Longitudinal OCTA monitoring revealed accelerated regression of hyaloid vessels correlated with retinal degeneration in rd10. Quantitative OCT measurement disclosed significant distortions of both retinal thickness and the vitreous chamber in rd10 compared to WT mice. These OCT/OCTA observations confirmed the close relationship between HVS physiology and retinal neurovascular development. The distorted HVS regression might result from retinal hyperoxia or dopamine abnormality due to retinal remodeling in rd10 retina. By providing a noninvasive imaging platform for longitudinal monitoring of HVS regression, further OCT/OCTA study may lead to in-depth understanding of the physiological mechanisms of HVS regression in normal and diseased eyes, which is not only important for advanced study of the nature of the visual system but also may provide insights into the development of better treatment protocols of congenital eye disorders.

## Introduction

The hyaloid vascular system (HVS) plays an important role in nourishing immature intraocular tissues during embryonic development of the eye^1^. The HVS undergoes spontaneous regression that coincides with the development of trilaminar retinal vascular plexuses in the fetus by 34 weeks of gestation^2^. Failure of HVS regression can result in a congenital anomaly of the eye; i.e., persistent fetal vasculature (PFV) that leads to intraocular hemorrhage, retinal detachment, and cataract^3–5^. There are more than 450,000 preterm deliveries in the United States each year^6^, accounting for >10% of the ~3.9 million newborns annually^7^. More than 90% of premature infants have hyaloidal vascular remnants present at birth; these hyaloid remnants typically regress by the time maturity is reached or a few weeks later^8^. In-depth knowledge of the HVS physiology is thereby essential for a better understanding of developmental eye disorders as well as for optimal treatments to effectively prevent vision loss of premature infants.

However, the physiological mechanisms of HVS regression within the framework of ocular development are still elusive due to technical limitations of conventional fixed tissue analysis^9,10^. Histological examination only provides end-point snapshots of the complex HVS remodeling. Thus, the natural context of HVS physiology can hardly be appreciated with the histological technique, and the shapes of postmortem eyeballs can significantly alter within a few minutes^11^. Experimental interventions of living fetuses and infants are technically challenging as well. Further investigation with animal models may provide insights that elucidate HVS regression mechanisms in the developing eye.

There are active efforts to pursue *in vivo* monitoring of HVS dynamics in animal eyes. Microvascular corrosion casting and microcomputed tomography (μCT) have been combined to demonstrate three-dimensional (3D) imaging of the HVS in neonatal mice^12^. Scanning laser confocal microscopy has been used to visualize intraocular vasculatures including the HVS^13^. High frequency ultrasound biomicroscopy (UBM) of postnatal mouse eyes revealed a progressive decrease in blood flow velocity in the HVS with aging^14^. Larina *et al*. developed an *in utero* embryonic ocular imaging method utilizing optical coherence tomography (OCT) for phenotypic analysis of intraocular structures, and they successfully visualized some of the HVS in mouse embryos^10^. As a new OCT modality, OCT angiography (OCTA) can provide quantitative imaging of functional ocular vasculatures. Recently, we demonstrated for the first time the feasibility of *in vivo* OCT/OCTA imaging of HVS regression in immature mouse eyes. OCT enabled morphological monitoring of the HVS structure, while OCTA allowed functional assessment of the hyaloid vessels^15^.

The present study demonstrates longitudinal OCT/OCTA monitoring of HVS regression in the retinal degeneration 10 (rd10) mice in which rod photoreceptors die during ocular development. Rd10 carries a spontaneous missense point mutation in PDE6B (cGMP phosphodiesterase 6B, rod photoreceptor)^16^. Progressive rod cell death starts at around postnatal day (P) 16, at which time HVS regression is still in progress^16^, which allows correlating HVS regression with ocular structural deformities. Comparative OCTA study of wild-type (WT) and rd10 mice has also revealed a severe retinal deep capillary plexus (DCP) malfunction tightly correlated with the onset of photoreceptor degeneration^17^. Since disturbed retinal development may affect the persistent hyaloid vessels^3^, we expect that rd10 mice would manifest a different HVS regression pattern compared to WT mice.

## Results

**Figure 1:**
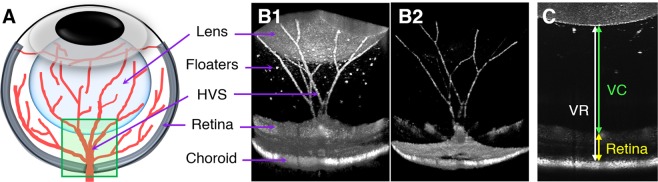
(**A**) Schematic diagram of the hyaloid vascular system (HVS) in the mouse eye. The green box indicates the OCT/OCTA imaging area. Representative (**B1**) OCT and (**B2**) OCTA. In this imaging field, the hyaloid vessels can be clearly captured together with neighboring ocular tissues including the lens, the retina, and the vitreous with floaters. (**C**) Axial length measurements of the posterior segment. Vitreoretinal (VR) length (white line), vitreous chamber (VC) length (green line) and retinal thickness (yellow line) were separately measured.

Three-dimensional OCT/OCTA images of the posterior segment (Fig. 1A) were acquired longitudinally in WT and rd10 mice. Representative volumetric OCT and OCTA images are shown in Fig. 1B. This imaging field contained the posterior pole of the lens, vitreous, optic nerve head (ONH), retina, and HVS with floaters. The axial length of the posterior chamber as well as the retinal thickness were measured to analyze a potential correlation with HVS regression (Fig. 1C).

### Progressive retinal degeneration begins at P17 in rd10 mice

**Figure 2:**
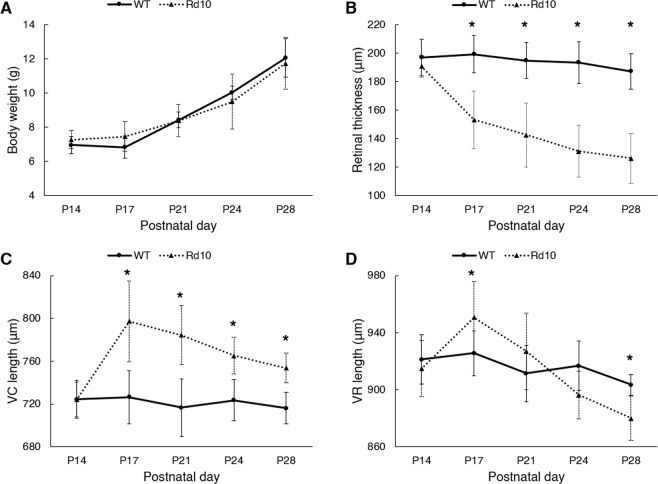
(**A**) Body weight measurement. (**B**) Retinal thickness measurement. Photoreceptor degeneration resulted in significant thinning of the retinal thickness in rd10 mice: 190.8 ± 7.6 μm (P14), 153.4 ± 20.2 μm (P17), 142.6 ± 22.3 μm (P21), 131.1 ± 18.2 μm (P24), and 126.3 ± 17.5 μm (P28). (**C**) VC length measurement. Onset of photoreceptor degeneration induced rapid extension of the VC length (724.0 ± 16.3 μm [P14] to 797.3 ± 38.0 μm [P17]), indicating collapse of the inner retinal layers. Subsequently, the VC length of rd10 mice steadily decreased with aging: 784.3 ± 27.5 μm (P21), 765.3 ± 17.1 μm (P24), and 753.7 ± 14.1 μm (P28). WT showed little change in the VC length throughout the experimental period: 724.3 ± 17.7 μm (P14) to 716.2 ± 14.7 μm (P28). (**D**) VR length measurement. Rd10 mice initially had a widened VR length in response to the onset of photoreceptor degeneration (914.8 ± 19.7 μm [P14] to 950.7 ± 25.3 μm [P17]), followed by a sharp narrowing phase (926.9 ± 26.8 μm [P21] to 880.0 ± 15.5 μm [P28]). The VR length in WT mice showed a slight declining trend (921.2 ± 17.3 μm [P14] to 903.4 ± 7.2 μm [P28]). *N* = 7 for WT and *N* = 7 for rd10. *P* values for an unpaired t-test between WT and rd10 mice are indicated by asterisks: *P < 0.05.

We first compared the body weights of WT and rd10 mice (Fig. 2A). Checking body weight is important rather than simply referring to the chronological age of juvenile animals because the growth rate of the eye is highly correlated with the growing body size^18–20^. At P14, the mean body weight of WT and rd10 mice was 6.97 ± 0.5 g and 7.28 ± 0.5 g, respectively. The body weight steadily increased with aging and reached 12.05 ± 1.1 g in WT mice and 11.74 ± 1.5 g in rd10 mice at P28. No significant variation was found between the two genotypes at each time point.

Unlike the incremental body growth, retinal thickness was significantly reduced in rd10 mice at P17 (199.4 ± 13.1 μm [WT] to 153.4 ± 20.2 μm [Rd10], *P* = 0.0003) as shown in Fig. 2B. Retinal thinning is a direct indicator of photoreceptor cell death, and it is mainly attributed to a decline in the outer retinal thickness due to photoreceptor degeneration^17^. Considering no significant difference in the retinal thickness at P14 between the two groups, the onset of rod photoreceptor cell death was expected to occur after P14 and before P17. The retinal thickness of WT mice slightly decreased with aging.

### Onset of photoreceptor cell death significantly altered the posterior segment

The axial length of the vitreous chamber actively changes under the interplay of genetics and environmental factors during ocular development through a process called emmetropization^21^. We noted that the axial length of the vitreous abruptly changed in rd10 mice in conjunction with retinal thinning. For comparison, the vitreous chamber (VC) length, from the posterior pole of the lens to the retinal nerve fiber layer (RNFL), and the vitreoretinal (VR) length, from the posterior pole of the lens to the retinal pigment epithelium (RPE), were measured separately (Fig. 1C). At P14, the VC length was not significantly different between WT and rd10 mice, as shown in Fig. 2C (721.5 ± 24.7 μm [WT] and 724.0 ± 16.3 μm [Rd10]). However, the VC length of rd10 mice at P17 dramatically increased in response to the onset of photoreceptor cell death (797.3 ± 38 μm in rd10, 726.3 ± 24.7 μm in WT [*P* = 0.0014]), indicating that the inner retinal layers collapsed to the RPE side due to the degenerating photoreceptor layer. Thereafter, the VC in rd10 mice quickly narrowed the gap between the lens and the retina as the VR length started to decrease with aging. As shown in Fig. 2D, after the onset of photoreceptor degeneration, the VR length of rd10 mice also transiently increased to 950.7 ± 25 μm at P17 from 914.8 ± 19.7 μm at P14; then it rapidly decreased and reached 880 ± 15.5 μm at P28, which was even much shorter than that of WT mice (903.4 ± 7.2 μm) at P28 (*P* = 0.0036). These abnormal changes of the posterior segment of the eye indicate an active emmetropization process in rd10 mice, adjusting the axial length of the eye to the focal plane of the ocular optics^22^.

### Functional OCTA reveals accelerated regression of hyaloid vessels in rd10 mice

**Figure 3:**
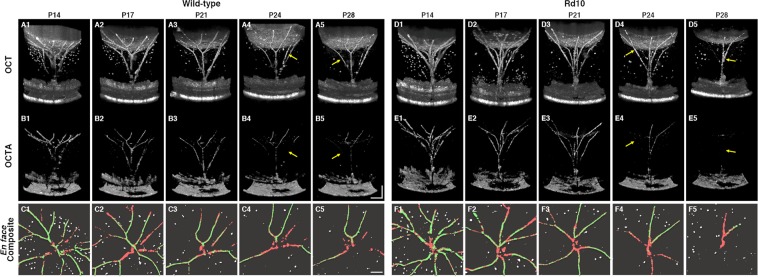
Representative (**A,D**) OCT and (**B,E**) OCTA volumetric images from a WT mouse and an rd10 mouse, longitudinally recorded at P14, P17, P21, P24, and P28. In binarized *en face* composite images (**C**,**F**), red, white, and green colors represent the hyaloid vasculature, floaters, and functional vessels, respectively. The retinal and choroidal vasculature was excluded from *en face* image construction. Yellow arrows indicate capillaries in functional loss; i.e., a lack of blood flow. *Scale bars*: 200 μm.

Figure 3 illustrates representative OCT/OCTA datasets from a WT mouse and an rd10 mouse throughout the longitudinal observations. Unlike the retinal vasculature embedded in densely packed retinal tissues, the hyaloid vessels are surrounded by a transparent structure, making OCT itself available to image the HVS. In addition, OCT enables capturing numerous hyperreflective round-shaped floaters in the vitreous, which are presumably macrophages^23^. However, OCTA provides enhanced contrast of the vascular structure and it also preserves functional information. In the OCTA B-scan images (Fig. 3B,E), the contrast enhanced hyaloid vasculature is clearly emphasized. For quantitative comparison, binarized *en face* images were prepared, and the ‘vascular structure’ density was differentiated, as processed with the OCT dataset, from the ‘functional vessel’ density, as processed with the OCTA dataset. In *en face* composite images (Fig. 3C,F), the binarized vasculatures, floaters, and functional vessels are presented in a red color, white color, and green color, respectively. It can be easily recognized in Fig. 3 that the numbers of vascular branches and floaters decreased with aging in both groups.

**Figure 4:**
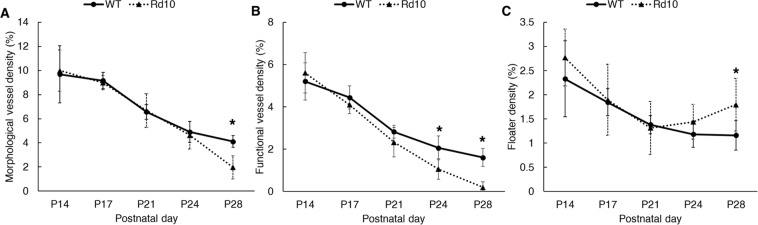
(**A**) Hyaloid vasculature density measurements from OCT datasets. Accelerated regression of hyaloid vessel occurred in rd10 mice compared to WT mice (4.11 ± 0.5% [WT at P28] and 1.98 ± 0.9% [Rd10 at P28], *P* = 0.0002), indicating a functional link between retinal degeneration and hyaloid regression during eye development. (**B**) Functional hyaloid vessel density measurements from the OCTA datasets. Functional OCTA revealed even earlier physiological dysfunction in rd10 mice before the morphological regression of the hyaloid vasculature (2.06 ± 0.6% [WT at P24] and 1.05 ± 0.5% [Rd10 at P24], *P* = 0.0061). (**C**) Floater density measurements. Floater density steadily decreased in both WT and rd10 mice with aging; however, its population escalated at P28, which coincided with the rapid vessel dropout in rd10 mice (1.16 ± 0.3% [WT at P28] and 1.8 ± 0.5% [Rd10 at P28], *P* = 0.0003); *N* = 7 for WT and *N* = 7 for rd10. *P* values for an unpaired t-test between WT and rd10 mice are indicated by asterisks: *P < 0.05.

In quantitative analysis, the morphological vessel density (Fig. 4A) and floater density (Fig. 4C) were not significantly different between the two groups until P24. However, rd10 mice showed a rapid vascular dropout at P28, resulting in an almost vessel free VC (1.96 ± 0.9%); while the WT showed a modest declining trend in vessel density, and several vessels remained functional even at P28 (4.11 ± 0.5%). Following the advanced vascular regression in rd10 mice, the floater density also sharply increased at P28 (*P* = 0.0003). Intriguingly, before the vessel fragments, a significant functional impairment was first noted in rd10 mice at P24; i.e. a lack of blood flow (Fig. 4B). These results demonstrate that retinal degeneration in eye growth ultimately accelerates the HVS regression process.

### Cardinal direction dependent regression rates of the HVS

**Figure 5:**
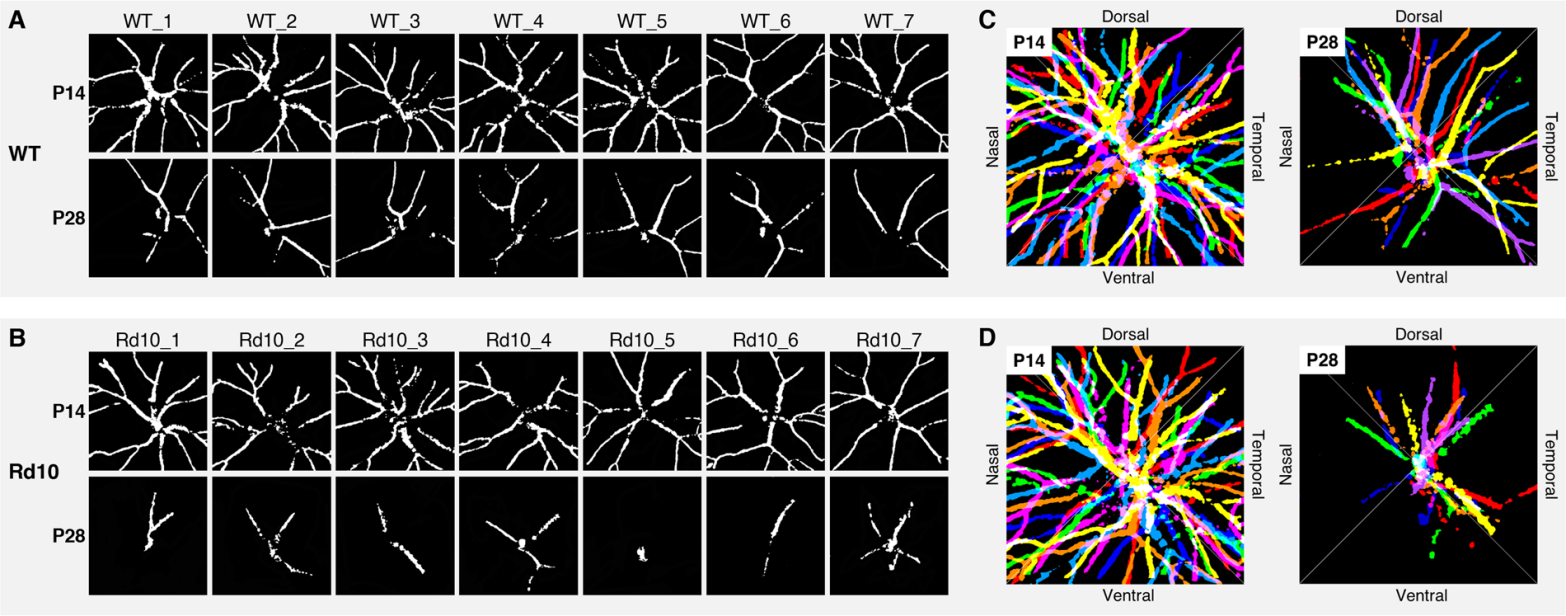
Binarized *en face* image sets of hyaloid vasculatures obtained from (**A**) 7 WT and (**B**) 7 rd10 mice at P14 and P28. (**C**,**D**) Represent superimposed images over 7 different binarized vessel images from each set of (**A**) WT and (**B**) rd10. Seven colors of the rainbow are painted for intuitive visualization.

We were also aware of the asymmetric distribution of the persistent hyaloid vessels at P28 (Fig. 5). While the hyaloid vessel distribution was quite homogeneous in both groups at P14, the remaining vessels at P28 were mostly within the dorsal and temporal areas in both WT (Fig. 5C) and rd10 mice (Fig. 5D), indicating different regression rates on the cardinal axes. Given the factor that WT and rd10 groups revealed this identical atrophic pattern, they may share a similar signaling pathway but in different tempos.

## Discussion

Longitudinal OCT and OCTA measurements were used to monitor HVS regression in the developing mouse eyes from P14 to P28. Following the onset of photoreceptor degeneration, accelerated functional and morphological regression of the HVS was confirmed in rd10 mice. This premature hyaloid regression is a unique case, along with recently investigated opsin-5 null mice, since most mutant mice have shown persistent hyaloid vessels if the DCP formation is disturbed^24^. In other words, the DCP in rd10 mice can be considered fully vascularized before the onset of photoreceptor cell death. On the contrary, the WT mice revealed that several hyaloid vessels lasted over 4 weeks of age, which may imply that extant functional hyaloid vessels still intervene with eye growth.

Considering the rationale that the HVS transiently nurtures the growing ocular components, the remaining functional HVS in the WT mice could be involved in later developmental stages of the ocular components, and the accelerated regression of hyaloid vessels in rd10 mice might partially limit ocular development. Multiple studies have reported that rd10 mice have lighter eye weight and shorter axial length of the eyeball than WT mice^25,26^. In fact, mice eyes continuously grow up to P300, but the growth rate splits into two or three distinctive phases^27–29^. The initial phase, up to P40, is a period of rapid growth and it is characterized by very rapid enlargement of the eyeball, and later phases are characterized by slow expansion of the eyeball^28^. Since almost all hyaloid vessels of rd10 mice regressed during the initial growing step, we can plausibly postulate that the immature HVS regression may impinge on the subsequent eye developmental stages.

Although the exact triggering mechanism responsible for HVS regression is not fully understood, multiple contributing factors are simultaneously associated with the regression process, such as apoptosis of endothelial cells followed by phagocytosis of the atrophic vessels^30,31^ and vasoconstriction of the hyaloid vessels due to reduced blood flow^32^. HVS regression is also considerably under the influence of retinal oxygen levels that directly regulate the expression of vascular endothelial growth factor (VEGF), a signaling protein involved in the formation of hyaloid vessels as well as their maintenance^33^. A study showed that low oxygenation in the retina due to poor retinal vascularization induced high VEGF levels, consequently resulting in persistent hyaloid vessels^34^.

On the contrary, high oxygen levels lead to downregulation of VEGF levels that can accelerate HVS regression^35^. A study confirmed that inactivating VEGF proteins accelerated hyaloid vessel regression^33^. Given the identical atrophic pattern in the cardinal directions between the two genotypes and the accelerated pace in rd10 mice, the retinal hyperoxia in rd10 mice may play a crucial role in HVS regression. Rd10 mice showed rapid attenuation of the DCP as soon as rod photoreceptor death was initiated^17^, which naturally inflated oxygen diffusion towards the inner retina due to the choroid circulation that lacks autoregulating capacity in response to oxygen levels^36^.

Intriguingly, we also found that the persistent hyaloid vessels mostly rested on the dorsal/temporal areas in both WT and rd10 mice. A previous study noted that, in 12 adult mouse eyes with hyaloid arteries, all were found in the dorsal area^37^. One of the potential reasons may be the asymmetric retinal cell density. Although the mouse retina does not have a fovea, it does have an area of increased density of ganglion cells and photoreceptors in the dorsal area^38–41^. Since the oxygen level in the retina is a factor in HVS regression, the relatively hypoxic condition that is expected in the area with higher cell density, may contribute to the slow hyaloid regression in the dorsal/temporal areas. A previous study found a higher vascular density in the temporal hemisphere of the DCP compared to that of the nasal hemisphere, which can also support this notion^37^.

Neurotransmitter dopamine released by dopaminergic amacrine cells (DACs) in the retina can directly inhibit VEGF receptor-2 (VEGFR-2) signaling in hyaloid vascular endothelial cells and accordingly accelerate HVS regression^24^. Following the massive loss of photoreceptors, the inner retinal neurons undergo extensive remodeling in rd10 mice and this accompanies structural and physiological changes of DACs that cause abnormalities in dopamine metabolism^42^. A study also found that the retinal dopamine levels were significantly higher in rd10 mice compared to WT mice^26^. Interestingly, our data demonstrated that a functional hallmark of hyaloid regression in rd10 mice; i.e. a lack of blood flow, appeared at a relatively later time point; that is P24 and not at P17, in which the remarkable retinal thinning as well as significant degeneration of the DCP occurred^17^. Taken together, the modulated dopamine levels resulting from the inner retinal remodeling can be another potential triggering factor of accelerated hyaloid regression in rd10 mice.

Another potential reason for a small eye phenotype in rd10 mice may be the altered axial length of the vitreous that is tightly linked to the refractive power of the eye^28,43^. The mouse eye has an emmetropization mechanism and responds with myopic shifts when exposed to form deprivation or negative lens defocusing^11,44,45^. A serendipitous observation in our study was that rd10 mice showed a strong hyperopic shift in response to retinal degeneration, which shortened the axial length of the vitreous. According to the optical structure of the mouse eye, a refractive change of 1 diopter (D) is equivalent to 5.4–6.5 μm changes in the vitreous length^29^. Given the dynamic range of the observed VR length is about 70 μm in rd10 mice, it is equivalent to an approximate 12 D hyperopic shift within 2 weeks. Surprisingly, this is a huge and brief change and may provide a vital clue to previous studies finding significant hyperopia in rd10 adult mice^25,26,46^. While a myopic shift is characterized by elongation of the eyeball, a hyperopic shift limits the elongation of the eyeball by thickening the choroid through a signaling pathway from the RPE to the sclera^22,47^. Therefore, the strong hyperopic shift in rd10 mice can also contribute to a smaller eye phenotype. It is also well known that retinal dysfunction can be directly involved in the deregulation of eye development, and refractive errors are very common in patients with retinopathy of prematurity (ROP)^48^.

We also paid attention to the collapse of the inner retinal layers of rd10 mice at P17, immediately after the massive photoreceptor cell death. This collapse, along with the abrupt VR length increment, corresponds to a transient myopic shift; i.e., the distance from the lens to the retina suddenly increases. It is known that photoreceptor dystrophy is associated with myopia^19,49^, and the incidence of myopic refractive errors in retinitis pigmentosa (RP) patients is 75%, while patients with the X-linked form of RP have refractive errors that reach 95%^50^. The position shift of the inner retina, resting on the RPE side, may provide an interesting clue to understanding the rationale behind a myopic shift occurring in patients with photoreceptor degeneration.

In summary, our comparative OCT and OCTA study revealed accelerated regression of HVS in rd10 mice during ocular development, which may denote a key role of the VEGF expression levels modulated by either altered retinal oxygen concentrations or dopamine levels. Our results clearly indicate a close functional link between the retinal neurovascular system and the HVS. The present study illustrated well how a minute mutation in rod photoreceptor cells has momentous effects on the landscapes of the back of the eye. Concurrent OCT/OCTA study of HVS regression within the framework of ocular development using various mutant mouse models will further our understanding of developmental eye disorders.

## Methods

### Animal preparation

Seven WT mice (C57BL/6J, The Jackson Laboratory) and seven rd10 mice (homozygous for the Pde6b^rd10^ on C57BL/6J background; The Jackson Laboratory) were used in this study. These two strains have been extensively used for comparative study of morphological and physiological changes due to retinal degeneration^17,51–56^. Concurrent OCT and OCTA measurements were longitudinally conducted at P14, P17, P21, P24, and P28. During the experiment, mouse body weight was first measured for anesthetic dose estimation. Anesthesia was intraperitoneally induced by a mixture of 100 mg/kg ketamine and 5 mg/kg xylazine. A drop of 1% atropine sulfate ophthalmic solution (Akorn, Lake Forest, IL) was applied to the left eye where imaging was performed. The mouse was then moved to a custom-designed mouse holder, and a cover glass (12-545-80; Microscope cover glass, Fisherbrand, Waltham, MA) with a drop of lubricant eye gel (Severe; GenTeal, Novartis, Basel, Switzerland) was placed on the imaging eye. Once the mouse was fully anesthetized, the head was fixed for imaging acquisition.

All animal care and experiments were performed in accordance with the Association for Research in Vision and Ophthalmology statement for the use of animals in ophthalmic and vision research. All experiments were performed following the protocols approved by the Animal Care Committee at the University of Illinois at Chicago.

### OCT/OCTA volume acquisition

A custom-designed spectral domain OCT system was used for this study. A wide bandwidth near-infrared superluminescent diode (D-840-HP-I, Superlum, Cork, Ireland) with a central wavelength of 840 nm and a bandwidth of 100 nm was used as the light source, which provided ~3 µm axial resolution. The lateral resolution of the OCT system was estimated at 12 µm. A linear CCD camera (AViiVA EM4; e2v Technologies, Chelmsford, UK) was used in the OCT spectrometer, which provided a 30 kHz A-scan rate. The incident light power on the cornea was 0.95 mW. OCT/OCTA volumes were acquired over the area 1.2 mm (width) × 1.2 mm (length) × 1.4 mm (height), containing the posterior surface of the lens, the HVS, and the retina, as shown in Fig. 1B. The ONH was used as a reference imaging point. Four consecutive OCT B-scans were obtained at each recording position to calculate speckle variances (SV) for OCTA construction^57^. Thus, a total of 4 × 500 × 500 A-scans was acquired for each OCTA volume image.

### Retinal thickness and vitreous chamber length measurements

Retinal thickness, VR length, and VC length were separately measured, as shown in Fig. 1C. The retinal thickness was measured from the RNFL to the RPE. The VR axial length was measured from the posterior pole of the lens to the RPE, and the VC axial length was measured from the posterior pole of the lens to the RNFL. A B-scan approximately 120 μm dorsally away from the central ONH was used for this measurement, since the boundary of the retina is vague at the ONH. Triplicate measurements were manually implemented per each dataset to minimize observation error.

### Vessel and floater density analysis

**Figure 6:**
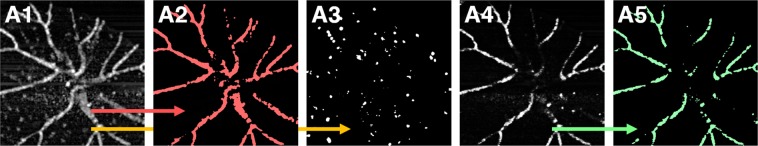
Image processing diagram. Constructed *en face* OCT (**A1**) and OCTA (**A4**) images for morphological and functional vessel density analysis, respectively. Applied image processing ultimately binarizes (**A2**) hyaloid vasculatures, (**A3**) floaters, and (**A5**) functional hyaloid vessels for density measurements.

In this study, we differentiated ‘morphological vessel’ density processed with the OCT dataset from ‘functional vessel’ density processed with the OCTA dataset. Figure 6 illustrates the image processing scheme for the vessel density analysis as well as floater density analysis. Floaters are mainly composed of macrophages and vessel fragments floating in the vitreous.

For the morphological vessel analysis, the 4 repeated OCT B-scans at each scanning position were first averaged. Second, a stack of OCT B-scans was sliced parallel to the B-scan cross-section to generate a C-scan (*en face*) stack, followed by contrast adjustment to individual C-scan images. Third, maximum intensity projection (MIP) was implemented in the C-scans (Fig. 6A1). The retinal vasculature was excluded in the projection. Fourth, binarization was realized with the Otsu’s automatic thresholding method^58^. Fifth, after binarization, floaters were manually removed (Fig. 6A2). Binarized floater images were also constructed following a similar procedure to the vasculature analysis, but before the binarization, the vascular structure was first removed, followed by Otsu’s automatic thresholding (Fig. 6A3).

For functional vessel analysis, OCTA images were first constructed by computing SV from 4 repeated OCT B-scans^57^. Second, a stack of OCTA B-scans was sliced parallel to the B-scan cross-section to generate a C-scan stack. Third, the MIP was implemented in the OCTA C-scans (Fig. 6A4). Fourth, binarization was realized by Otsu’s automatic thresholding method, followed by small particle-like noise removal (Fig. 6A5). All the image processing was performed with Fiji software (http://fiji.sc/Fiji).

### Statistical analysis

The density value was defined as the percentage of pixel area occupied by the target objects in the binarized projection image. All data are expressed as the mean ± standard deviation (SD) per group. Unpaired t-tests between the WT and rd10 groups at each age were performed using either Student’s t-test (equal variances) or Welch’s t-test (unequal variances), and a *P-value* < 0.05 was considered statistically significant.

## Data Availability

The other data that support the findings of this study are available from the corresponding author on reasonable request.
